# A Case of Refractory Delirium Managed by a Multimodal Approach Using Atypical Antipsychotics, Antidepressants, and Benzodiazepines in an ICU Setting

**DOI:** 10.7759/cureus.23354

**Published:** 2022-03-21

**Authors:** Koteshwareddy Vadagandla, Patlolla Sridhar Reddy, Sheen Antony, Patlolla Prasanthy Reddy

**Affiliations:** 1 Critical Care, Medicover Hospital, Hyderabad, IND; 2 Internal Medicine, Suburban Community Hospital, East Norriton, USA; 3 Registrar, Medicover Hospital, Hyderabad, IND

**Keywords:** benzodiazepine use in delirium, multimodal therapy, antidepressants, atypical anti-psychotic, persistent delirium

## Abstract

Delirium is an acute confusional state, most commonly observed in elderly patients admitted to the critical care unit. In most cases, early recognition, avoiding triggering factors and conservative measures are adequate for the management, but sometimes symptoms persist despite adequate medical care, which goes in the favor of refractory delirium. Refractory delirium has no clear-cut definition but it is discussed in some of the case reports and literature as the presence of symptoms despite adequate treatment without impairing consciousness. Management of such refractory symptoms requires careful evaluation to identify the cause and predominant symptoms, which further helps in choosing a better therapeutic regime. It is often difficult to manage such cases and require sedatives and anti-psychotics to reverse the condition. Atypical antipsychotics are now playing a prominent role in the management of refractory delirium, and the selection of a drug that is suitable for the patient profile with negligible side effects is of utmost importance. We are presenting one such case, with multiple causes for his delirium, with a predominant hyperactive state and the refractory symptoms managed by atypical antipsychotics, antidepressants, and benzodiazepines.

## Introduction

Delirium is the most common clinical condition observed in the ICU, which often goes unrecognized and may sometimes lead to increased morbidity and mortality, especially in the older population. According to the Diagnostic and Statistical Manual of Mental Disorders, Fifth Edition (DSM 5) criteria, the diagnosis of delirium requires four features: it should be acute in onset with alteration in attention, change in cognition, and associated with a direct link to a causative factor [1]. Based on the predominant feature with which it presents, delirium can be subdivided into three subtypes - hyperactive, hypoactive, and mixed variety. There are many theories to define the pathophysiology of this confusional state [2], but the two broadly circulating theories that gained popularity are - the Neurotransmitter Theory, which states the imbalance between excitatory and inhibitory neurotransmitters [3], and the Neuroinflammatory Theory, which briefs the role of cytokines in the pathology of delirium [4]. This acute confusional state is caused by a variety of factors, including advanced age, sepsis, abnormal metabolic parameters, polypharmacy, intoxication, and drug withdrawals. Many tools have been formulated to diagnose this condition among which the most commonly used tool in the ICU is the Confusion Assessment Method for the Intensive Care Unit (CAM-ICU) scale [5]. Early diagnosis and intervention give better outcomes for delirium, which can be managed by starting with conservative measures like counselling, spending time with family, ambulation, maintaining good sleep hygiene, and avoiding unnecessary procedures like insertion of Foleys catheter and feeding tubes [6]. In the case of established delirium, which cannot be managed by conservative measures, various classes of drugs have been used based on the subtype and associated causative factors such as benzodiazepines, antipsychotics and antidepressants [7]. Benzodiazepines are most commonly used for alcohol withdrawal [8], whereas antipsychotics are used in other types of delirium in acute settings. Atypical antipsychotics have taken a major role in the present-day treatment of delirium, especially in refractory cases, and the recommendations regarding usage of these drugs were reviewed in the literature by Boettger and Breitbart [9]. Even though there is no exact definition of refractory delirium, it has been broadly described as a symptom that persists despite the use of all other potential and acceptable symptomatic therapies that do not impair consciousness [10]. We are presenting a case of an elderly male with refractory delirium suspected to have multiple causes admitted to ICU. In this case, we have used a multimodal approach with benzodiazepines, atypical antipsychotics, and antidepressants to treat his refractory symptoms.

## Case presentation

A 70-year-old male patient presented to the hospital with complaints of throat pain and difficulty in swallowing for 2 months, associated with loss of weight and loss of appetite. He had a history of chronic smoking and alcohol abuse. On further evaluation, he was diagnosed with carcinoma oropharynx and admitted for radical radiotherapy and chemotherapy.

After the first session of radiotherapy and chemotherapy with carboplatin and nimotuzumab, he became agitated and restless and required multiple sedatives such as haloperidol 2.5 mg IV, risperidone 1 mg oral, and midazolam 2 mg IV to sleep. The patient however continued to remain agitated and restless and he was shifted to the ICU for further evaluation and management.

**Figure 1:**
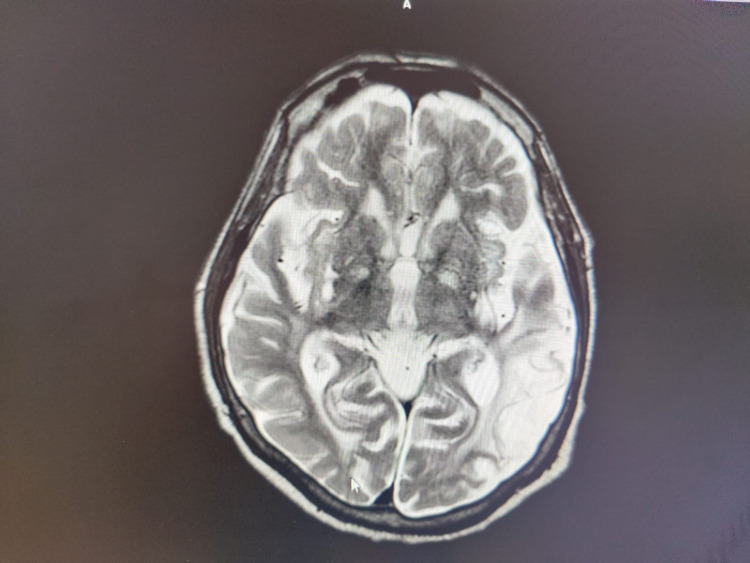
T2W MRI brain plain axial view Gliotic changes and cystic encephalomalacia at left parietooccipital lobes with loss of parenchymal volume. T2W MRI: Transverse relaxation time magnetic resonance imaging

All necessary investigations to rule out metabolic derangement and sepsis turned out normal (Table 1). MRI Brain showed gliotic changes and cystic encephalomalacia at left parietooccipital lobes with loss of parenchymal volume, as shown in* *Figure 1. In the ICU he developed extrapyramidal side effects (EPS) like rigidity, dyskinesia, hyperprolactinemia, in view of which haloperidol and risperidone were stopped and quetiapine 50 mg (OD) was added on the neurologist’s advice, as it has lesser extrapyramidal side effects in comparison with haloperidol.

**Table 1:** Summary of investigations BU- blood urea, Na- sodium, ca- calcium, Mg- magnesium, K- potassium, TSH- thyroid-stimulating hormone.

Complete Blood Picture	Insignificant, within normal limits
Renal function test	BU-24, Serum Creatinine - 1.03
Liver function	Insignificant, within normal limits
Electrolytes (Ca, Mg, Na, K)	Ca- 8.7, Mg -2.3, Na - 132, K -2.4
Thyroid function	TSH- 4.5
Prolactin	41.27
Procalcitonin	0.159
EEG	Minimal diffuse slowing
Blood and Urine cultures	Negative

After carefully evaluating his history, his delirium was quantified as multifactorial secondary to advanced age, tumour, chemotherapy, and alcohol withdrawal. The psychiatrist’s opinion was taken for further management. Upon conducting Mental Status Examination (MSE), he was found to be irritable, restless, agitated with disturbed sleep. Unsuccessful rapport was established as the patient was agitated. Inj. olanzapine 5 mg (HS [hour before sleep]) was added as it had an advantage of rapid onset with insignificant extrapyramidal side effects.

Keeping in mind the patient’s history of chronic alcohol abuse, withdrawal being one of the major factors contributing to his delirium, mirtazapine 7.5 mg (OD) was added to reduce his collateral depression and anxiety symptoms, along with thiamine 100 mg IV (OD) and benzodiazepines. Lorazepam 2 mg was given in the night and modafinil 100 mg was given in the morning to promote a better sleep-wake cycle. MSE and psychiatric evaluation were done daily, keeping the side effect profile of the antipsychotic medications in check.

The patient had continuous ECG abnormalities and hypotensive episodes with excessive daytime sleepiness and agitation in the night, due to which quetiapine was withheld as it was known to cause QT prolongation and drowsiness. Olanzapine was made SOS because of its predominant sedative action and poor tolerance in the older population. Aripiprazole 5 mg (OD) was added as it had proven efficacy in tumour psychosis with lesser side effects, along with opipramol 50 mg (OD) to reduce his anxiety. Naltrexone 50 mg (OD) was given as the patient had a history of chronic opioid use at home for tumour pain.

The patient was evaluated regularly by Mental Status Examination (Table 2). His final medications included aripiprazole, opipramol, naltrexone, mirtazapine, lorazepam at night and modafinil in the morning. With these medications on the chart, the patient's mental status was assessed daily and he was evaluated for any side effects of the drugs along with monitoring of other parameters. We noticed that over a period of 26 days the patient’s cognitive ability had improved, and psychomotor agitation and restlessness had resolved. His consciousness and orientation improved significantly by the 26th day, on the 27th day of therapy he started obeying commands, and by the 33rd day, he had started to communicate through gestures. We were able to establish a meaningful rapport with the patient. He was then shifted to the wards for further management.

**Table 2:** Mental state examination, sequence of clinical evaluation

Day of admission	Affect, consciousness, orientation	Motor	mood	Cognition	drugs	Speech and Clinical assessment
3	Irritable, asleep	Psychomotor agitation (night)	fluctuating aggression/depression	declined	Haloperidol SOS and Midazolam	Unsuccessful rapport, non-coherent.
4	Irritable, asleep	Psychomotor agitation (night)	fluctuating aggression/depression	declined	Haloperidol SOS, risperidone, Midazolam	Unsuccessful rapport, non-coherent
6	Irritable, asleep	Psychomotor agitatio(night)	fluctuating aggression/depression	declined	withheld risperidone, Haloperidol, started on Quetiapine and Mirtazapine.	Unsuccessful rapport, agitated.
9	Irritable, asleep	Psychomotor agitation (night)	fluctuating aggression/depression	declined	Quetiapine withheld, Mirtazapine and Olanzapine continued	Unsuccessful rapport, irrelevant speech.
11	Drowsy during the day, irritable during the night	Psychomotor agitation(night), restless	fluctuating aggression/depression	declined	Olanzapine and Mirtazapine continued.	Irrelevant speech.
12	Drowsy during the day, irritable during the night.	Psychomotor agitation(night) restless	fluctuating aggression/depression	declined	added Aripiprazole, Naltrexone and Opipramol	Agitated and irritable.
15	Drowsy during the day, irritable during the night.	Psychomotor agitation, restless	neutral	declined	Olanzapine made SOS, rest continued.	irrelevant speech.
18	Conscious and oriented, mild irritability.	Psychomotor agitation restlessness	neutral	better	Mirtazapine, Opipramol, Naltrexone, Aripiprazole	coherent and oriented to simple commands
24	Conscious, oriented, mild irritability, decreased discomfort	Psychomotor agitation, restlessness	neutral	better	Mirtazapine, Opipramol, Naltrexone, Aripiprazole	obeying simple commands
27	Conscious and oriented, wanting to communicate, clinically significant improvement found, obeyed commands.	Reduced Psychomotor agitation and restlessness	euthymic	improved	Mirtazapine, Opipramol, Naltrexone, Aripiprazole	Responding to commands.
33	Conscious and oriented, wanting to communicate, clinically significant improvement found, obeyed commands.	Reduced Psychomotor agitation and restlessness	euthymic	improved	Mirtazapine, Opipramol, Naltrexone, Aripiprazole	Patient was communicating via gestures. Successful rapport established.

## Discussion

It is prudent to distinguish between dementia, delirium, and other such acute neurological syndromes to have better management protocols and outcomes. According to DSM 5 criteria [1] (Table 3), there are clear-cut defining features that delineate this confusional state from other similar conditions. Delirium is an acute confusional state with many modifiable risk factors, although very common in the ICU setting, it often goes undiagnosed, resulting in augmented mortality and morbidity. This can present with a range of symptoms, based on which it is broadly classified as hyper, hypo, and mixed variety. Our patient was evaluated using CAM-ICU and was further diagnosed with mixed delirium with a predominant hyperactive state, with restlessness, agitation, and fluctuating sensorium. Risk factors for delirium are many among which some are predisposing, and others are triggering, of which advanced age, deranged metabolic parameters, sepsis, pain, dyselectrolytemia, polypharmacy, drug intoxication and withdrawal are some important causes to be considered [11]. In our case, delirium is multifactorial due to his age, tumour, chemotherapy, and history of alcohol dependence. Diagnosis of delirium is a challenging task as it has common manifestations with other neurological disorders such as dementia, depression, and other confusion syndromes. Many screening scales were devised to evaluate and diagnose this condition among which CAM-ICU was commonly used. We have screened our patient with the CAM-ICU scale (Table 3) and he has all four features, further confirming the diagnosis [5].

**Table 3:** DSM 5 Criteria to diagnose delirium and CAM ICU assessment scale DSM 5: Diagnostic and Statistical Manual of Mental Disorders, Fifth Edition; CAM-ICU: Confusion Assessment Method for the Intensive Care Unit

DSM 5 Criteria: Require all criteria to be met:
Disturbance in attention and awareness
Disturbance develops acutely and tends to fluctuate in severity
At least one additional disturbance in cognition
Disturbances are not better explained by a preexisting dementia
Disturbances do not occur in the context of a severely reduced level of arousal or coma Evidence of an underlying organic cause or cause
CAM-ICU assessment:
The presence of delirium requires features 1 and 2 and either 3 or 4
Acute change in mental status with a fluctuating course (feature 1)
Inattention (feature 2)
Disorganized thinking (feature 3)
Altered level of consciousness (feature 4)

Early management of delirium reduces morbidity and length of hospital stay in the elderly population. As mentioned by Pun et al in a cohort study, conventional measures like early mobilization, avoiding unnecessary interventions, providing adequate analgesia, treating early sepsis, correction of deranged electrolytes and other metabolic parameters mostly suffice and prevent further progression of the disease [12]. In established cases, where pharmacological measures play a crucial role and while selecting the drugs, two things should be given utmost importance: first being the identification of the causative risk factor and second being the classification of delirium into hyper, hypo, or mixed type for prompt correction and early recovery. Alcohol dependence should be carefully evaluated as the drug of choice in these cases are benzodiazepines. In our patient, the cause for delirium was multifactorial and he had a history of alcohol dependence, so we chose to give lorazepam 2 mg along with mirtazapine as it is a better drug to treat geriatric depression with collateral anxiety symptoms, secondary to alcohol dependence as seen in our patient [13].

The other class of drugs which are currently advancing in the management of delirium are antipsychotics which are broadly classified as typical and atypical based on their receptor selectivity, mechanism of action and side effect profile. They are associated with significant side effects, of which EPS effects are the most prominent, others being sedation, anticholinergic effects and cardiac instability [14]. So, the right drug is chosen based on the patient profile and their tolerance to the side effects. This has already been discussed in many studies. We used typical antipsychotics initially, like haloperidol (administered SOS) along with risperidone because of their rapid onset of action but later switched to other drugs as we noticed rigidity, finger fidgeting, tremors, and dyskinesia. Though risperidone tends to cause lesser EPS compared to haloperidol, which was observed by Han CS and Kim YK in their study [15], in our case it caused significant involuntary movements, so we switched to quetiapine, another first-line agent for delirium. However, after two days of quetiapine, we noticed cardiac instability in the patient, such as hypotension and arrhythmias forcing us to stop the drug which is a similar observation discussed by Bharadwaj and Slade in their case report [16].

Olanzapine was a better drug of choice in comparison to risperidone and quetiapine with better efficacy, quicker onset, lesser EPS and fewer cardiac complications. Our patient tolerated it initially, but we later noticed that it was causing more sedation, which was also described by Breitbart et al in their study [17], and a study comparing haloperidol with olanzapine gave the limitations of using olanzapine in the elderly population [18]. Because of these two drawbacks, this is the least favourable drug for hypoactive delirium and elderly patients. We continued olanzapine in this patient but administered it only on an 'as required' basis.

Aripiprazole, an atypical antipsychotic, was recently studied for refractory delirium and was proven to be better tolerated in the elderly and in cancer patients as discussed in their study by Boettger S and Breitbart W [19]. Aripiprazole has lesser EPS, better cardiac stability, least sedative action, no adverse effects on metabolic parameters and blood sugar levels. It provides a good safety profile, so was chosen further for our patient. After the initiation of this drug, the patient was closely monitored and daily MSE assessment was done which showed better progression in our patient with negligible side effects.

In our case, along with antipsychotics, opipramol was added to treat anxiety and naltrexone was added to counteract the opioid dependency, as he had a history of long-term opioid use (morphine) for tumour pain. In addition to this, mirtazapine was given to treat his collateral depression with anxiety [13], and to promote good sleep-wake cycle lorazepam was administered at night and modafinil was given in the morning. In addition to promoting wakefulness, modafinil is known to be beneficial in improving cognitive function which was reviewed by Minzenberg and Carter in their literature [20].With this multimodal approach, the patient showed an improvement in the overall neurological status and cognition with the least side-effect profile.

## Conclusions

Refractory symptoms in delirium are rare to occur and often difficult to treat. In such cases, careful evaluation to identify the cause and diagnosis of predominant symptoms help in choosing a better therapeutic regime. Atypical antipsychotics are often required to manage delirium if conservative measures fail but they are associated with significant side effects. So, choosing drugs should be individualized and careful monitoring should be done to avoid side effects. In our patient, based on his psychiatric evaluation, we used a multimodal approach with aripiprazole, mirtazapine, opipramol, and naltrexone, and maintained a good sleep-wake cycle by serving lorazepam at night and modafinil in the morning. Aripiprazole in our case provided a better recovery with fewer side effects when compared to haloperidol, risperidone and quetiapine. Mirtazapine is not regularly used to treat delirium, but in our case, we added it to manage the symptoms of collateral anxiety with depression as our patient had a history of alcohol dependence.
